# Transdisciplinary Community-Engaged Research Food Insecurity and Cardiovascular Health: Mobile Health Platform

**DOI:** 10.1093/geroni/igaf122.275

**Published:** 2025-12-31

**Authors:** Ana Diallo, Katherine Falls, Rachel Regal, Jered Wendte, Natalie Mansion, Leland Waters, Johnathan Williams, Kimberly Battle

**Affiliations:** Virginia Commonwealth University School of Nursing, Richmond, Virginia, United States; Virginia Commonwealth University, Richmond, Virginia, United States; Virginia Commonwealth University, Richmond, Virginia, United States; Virginia Commonwealth University School of Nursing, Richmond, Virginia, United States; Virginia Commonwealth University, Richmond, Virginia, United States; Virginia Commonwealth University, Richmond, Virginia, United States; Virginia Commonwealth University Center for Translational Research, Richmond, Virginia, United States; Virginia Commonwealth University, Richmond, Virginia, United States

## Abstract

The Mobile Health and Wellness Program (MHWP) serves as a platform for transdisciplinary community-engaged research (CEnR), aimed at driving innovations to address complex conditions associated with aging. In this forum, we share our experience using transdisciplinary approaches to tackle challenges older adults face, with research spanning food insecurity, cardiovascular disease, pharmacogenomics, Alzheimer’s research, and brain health interventions. One aspect of our research focused on food insecurity, we implemented a 6-12 week Wellness and Nutrition Education Program (PPP). The program included weekly or bi-weekly wellness sessions and provided participants with bags of fruits and vegetables. These sessions emphasized goal-setting as a tool for promoting behavior change. We conducted a pre-post descriptive analysis to assess the program’s impact on nutrition, food insecurity, and health measures such as blood pressure, blood glucose, and medication adherence. Eighty-one participants enrolled and 71 (88%) completed the program. On average, participants were 66.9 years old, and the majority identified as African American (79%) and women (70%), with 54% having a high school education or less. Participants reported an average of five chronic diseases, with hypertension (75%), type 2 diabetes (38%), and hypercholesterolemia (36%) being the most common. At baseline, 53% screened positive for food insecurity, and 51% reported nutrition insecurity. By completion, 92% of participants received six bags of fruits and vegetables. The program was significantly associated with an 18% decrease in nutrition insecurity (Z= -2.4, p = 0.02). These results highlight the effectiveness of the transdisciplinary CEnR model in addressing complex health challenges through collaboration.

